# RNA editing regulates glutamatergic synapses in the frontal cortex of a molecular subtype of Amyotrophic Lateral Sclerosis

**DOI:** 10.1186/s10020-024-00863-2

**Published:** 2024-07-12

**Authors:** Korina Karagianni, Dimitra Dafou, Konstantinos Xanthopoulos, Theodoros Sklaviadis, Eirini Kanata

**Affiliations:** 1 Department of Genetics, Development, and Molecular Biology, School of Biology, Aristotle University of Thessaloniki, 541 24 Thessaloniki, Greece; 2 Laboratory of Pharmacology, Department of Pharmacy, School of Health Sciences, Aristotle University of Thessaloniki, 54124 Thessaloniki, Greece; 3 Institute of Applied Biosciences, Centre for Research and Technology Hellas, 57001 Thermi, Greece

**Keywords:** Amyotrophic Lateral Sclerosis, Molecular subtype, ALS-Ox, RNA editing, Frontal cortex, Glutamatergic synapse

## Abstract

**Background:**

Amyotrophic Lateral Sclerosis (ALS) is a highly heterogenous neurodegenerative disorder that primarily affects upper and lower motor neurons, affecting additional cell types and brain regions. Underlying molecular mechanisms are still elusive, in part due to disease heterogeneity. Molecular disease subtyping through integrative analyses including RNA editing profiling is a novel approach for identification of molecular networks involved in pathogenesis.

**Methods:**

We aimed to highlight the role of RNA editing in ALS, focusing on the frontal cortex and the prevalent molecular disease subtype (ALS-Ox), previously determined by transcriptomic profile stratification. We established global RNA editing (editome) and gene expression (transcriptome) profiles in control and ALS-Ox cases, utilizing publicly available RNA-seq data (GSE153960) and an in-house analysis pipeline. Functional annotation and pathway analyses identified molecular processes affected by RNA editing alterations. Pearson correlation analyses assessed RNA editing effects on expression. Similar analyses on additional ALS-Ox and control samples (GSE124439) were performed for verification. Targeted re-sequencing and qRT-PCR analysis targeting CACNA1C, were performed using frontal cortex tissue from ALS and control samples (n = 3 samples/group).

**Results:**

We identified reduced global RNA editing in the frontal cortex of ALS-Ox cases. Differentially edited transcripts are enriched in synapses, particularly in the glutamatergic synapse pathway. Bioinformatic analyses on additional ALS-Ox and control RNA-seq data verified these findings. We identified increased recoding at the Q621R site in the GRIK2 transcript and determined positive correlations between RNA editing and gene expression alterations in ionotropic receptor subunits GRIA2, GRIA3 and the CACNA1C transcript, which encodes the pore forming subunit of a post-synaptic L-type calcium channel. Experimental data verified RNA editing alterations and editing-expression correlation in CACNA1C, highlighting CACNA1C as a target for further study.

**Conclusions:**

We provide evidence on the involvement of RNA editing in the frontal cortex of an ALS molecular subtype, highlighting a modulatory role mediated though recoding and gene expression regulation on glutamatergic synapse related transcripts. We report RNA editing effects in disease-related transcripts and validated editing alterations in CACNA1C. Our study provides targets for further functional studies that could shed light in underlying disease mechanisms enabling novel therapeutic approaches.

**Supplementary Information:**

The online version contains supplementary material available at 10.1186/s10020-024-00863-2.

## Introduction

Amyotrophic Lateral Sclerosis (ALS) is a heterogeneous (Grad et al. 2017), adult onset, neurodegenerative disorder, characterized by progressive deterioration of upper and lower motor neurons in the brain and spinal cord, resulting in muscle weakening, paralysis and ultimately death due to respiratory failure, typically within 2–5 years after disease onset. In addition to motor dysfunction, different degrees of cognitive and/or behavioural impairment occur in approximately 50% of ALS patients (Ringholz et al. 2005). ALS most frequently affects males and its incidence in Europe is estimated to 2–3 individuals per 100,000 (Hardiman et al. 2017).

ALS manifests as familial (fALS, 10–15%), associated with variations in more than 40 genes (Wang et al. 2023a), or sporadic (sALS, 85–90%). Genetic variation in ALS-related genes, such as C9orf72, SOD1, TARDBP, FUS, NEK1, OPTN, TBK1, ATXN1, ATXN2, NIPA1 and UNC13A, also occurs in a small percentage of sALS cases (Van Daele et al. 2023).

Neuropathological changes include atrophy, reactive astrogliosis and accumulation of cytoplasmic protein aggregates, mainly in the motor cortex and spinal cord. Accumulating evidence on the involvement of additional cell types, including immune system components, and brain regions in disease progression, has resulted in the current view that ALS is a multisystem neurodegenerative disease (Tortarolo et al. 2017; Grossman 2019). Structural and imaging studies support the involvement of the frontal cortex in ALS (Li et al. 2021; Pandya et al. 2022), and synaptic loss in the prefrontal cortex was found to be significantly higher in cognitively impaired cases (Henstridge et al. 2018).

At the molecular level, ALS displays characteristic traits of neurodegenerative diseases and its manifestation is dictated by interconnected molecular processes, regulated by complex networks (Mead et al. 2023; Wilson et al. 2023). Protein mislocalization/aggregation, RNA/DNA and synaptic defects have been suggested as crucially involved in ALS pathogenesis (Mead et al. 2023; Wilson et al. 2023).

Studies on post-mortem tissue from ALS patients provide the most direct source for unravelling disease related changes referring to pathology and underlying molecular mechanisms, while studies on in vivo or in vitro disease models allow significant advances in the understanding of specific pathogenetic aspects (Liguori et al. 2021; Hruska-Plochan et al. 2024). Despite the significant advances achieved up to date, detailed molecular mechanisms of disease progression are still elusive.

The advent of high-throughput -omics platforms and bioinformatics tools for analysis and integration of big data have greatly enhanced our understanding on ALS heterogeneity and underlying pathogenetic mechanisms. RNA editing is anticipated to extend our knowledge on the complex regulation of networks driving disease-related mechanisms.

RNA editing is a widespread co/post-transcriptional epigenetic mechanism, highly abundant in the human brain (Picardi et al. 2015). Its prevalent type refers to deamination of Adenosine to Inosine (A-to-I), mediated by the RNA Binding Protein Adenosine Deaminase Acting on RNA (ADAR) family members, ADAR1 and ADAR2. RNA editing is modified by neuronal activity and external stimuli (Balik et al. 2013; Licht and Jantsch 2016) and altered in neurological/neurodegenerative disorders, including ALS (Takuma et al. 1999; Kawahara et al. 2004; Flomen and Makoff 2011; Hideyama et al. 2012; Srivastava et al. 2017; Tran et al 2019; Moore et al. 2019; Kanata et al 2019; Ma et al. 2021; Dafou et al. 2022; Karagianni et al. 2022; Choudhury et al. 2023).

In this study, we investigated RNA editing profiles in the frontal cortex of a recently established ALS molecular subtype determined by transcriptome stratification analysis (Tam et al. 2019; Eshima et al. 2023). We focused on the most prevalent subtype (ALS-Ox), which presents characteristics of oxidative and proteotoxic stress, processes previously reported to contribute to neuronal degeneration (Mead et al. 2023; Wilson et al. 2023).

Our results unravel widespread RNA editing alterations and highlight modifications in transcripts involved in synaptic functions, specifically in the glutamatergic synapse pathway; we report altered recoding in a kainate receptor subunit, and by integrating transcriptome and editome profiles we identify differentially edited transcripts presenting altered expression. Our findings are verified through bioinformatics analyses on additional RNA-seq data from ALS-Ox and control cases and by experimental validation of RNA editing alterations associated with expression in the CACNA1C transcript. Our study extends previous knowledge on the role of RNA editing in ALS by reporting alterations in the frontal cortex of ALS-Ox cases, highlighting targets for further functional studies in disease models.

## Methods

### Data

We aimed to establish RNA editing profiles and investigate whether deregulated RNA editing is involved in disease-related processes in the frontal cortex of ALS-Ox cases. We analyzed a subset of FASTQ files included in the publicly available GSE153960 (Prudencio et al. 2020) data repository, generated through the New York Genome Center (NYGC) ALS Consortium (https://www.nygenome.org/). All selected ALS samples were assigned the most prevalent molecular ALS subtype, characterized by oxidative and proteotoxic stress (ALS-Ox), based on transcriptome stratification performed by Eshima et al. (2023).

The samples selected for analysis correspond to frontal cortex data from typical ALS cases (n = 45, Female: 16, Male: 29, Mean age at death: 64.2 ± 10.9 years), with no reported mutations in SOD1, C9orf72 (negative: 39, unknown: 6) or ATXN2 (negative: 31, unknown: 14). Typical ALS with concurrent other neurological disorders was reported for some of these cases (subject group ALS spectrum Motor neuron disease-MND/Other Neurological Disorders, n = 9).

As controls we used data from the frontal cortex of non-neurological disease donors (n = 48, Female: 21, Male: 27, mean age at death: 66.4 ± 10.8 years), who passed away due to cardiovascular (n = 16), neoplastic (n = 7), respiratory (n = 6), gastrointestinal (n = 5) or other reasons (urinary sepsis n = 1, unknown n = 13).

No significant differences were detected between the control and ALS groups in terms of age at death (Welch’s test p = 0.3463). Information on the analyzed samples is presented in Additional file 1. Table 1 summarizes ALS-Ox and control donor data.

**Table 1:** Summary of analyzed samples donor information

ALS cases Clinical Data (GSE153960)								
ALS molecular subtype (based on Eshima et al. 2023)	Gender	Age at onset (years)	Site of motor onset	Family history of ALS/FTD	Age at death (years)	Disease duration (months)	Subject group	Cause of death
ALS-Ox: n = 45	Female: 16 Male: 29	Range: 35–81 Average ± SD: 61.6 ± 11.1 (NA: n = 5)	Limb: n = 27 Axial and Limb: n = 1 Bulbar: n = 10 Bulbar and Limb: n = 2 NA: n = 5	Yes: n = 1 No: n = 2 NA: n = 42	Range: 38–83 Average ± SD: 64.2 ± 10.9	Range: 12–117 Average ± SD: 37.7 ± 22.1	ALS Spectrum MND*: n = 36 ALS Spectrum MND, Other Neurological Disorders**: n = 9	ALS/MND^$^: n = 14 Euthanasia: n = 6 Respiratory: n = 4 NA: n = 21

ALS cases available genetic data								
SOD1 mutation	C9orf72 expansions	ATXN2 expansions	Other					
Negative: n = 45	Negative: n = 39 Unknown: n = 6	Negative: n = 31 Unknown: n = 14	Positive for SETX, CCNF (likely benign): n = 1					

Control data (GSE153960)								
Non-neurological	Gender	Age at death (years)	Cause of Death					
Control: n = 48	Female: n = 21 Male: n = 27	Range: 44–89 Average ± SD: 66.4 ± 10.8	Cardiovascular: n = 16 Neoplastic: n = 7 Respiratory: n = 6 Gastrointestinal: n = 5 Other: n = 1 NA: n = 13					

MND: Motor Neuron Disease; NA: not available/unknown

*Classical/Typical ALS

**Classical/Typical ALS-AD (n = 8) & Classical/Typical ALS- FTD (n = 1)

^$^ALS/MND/ALS complications/MND complications/Respiratory ALS related/Respiratory MND related

For verification purposes, an additional set of RNA-seq data corresponding to 17 ALS-Ox and 6 control cases, retrieved from the publicly available dataset GSE124439, were also analyzed. Additional file 2 provides pertinent donor information from the GSE124439 dataset along with bioinformatics analysis results.

### Bioinformatics analysis

#### Read preprocessing, alignment and quality metrics

Raw RNA sequencing (RNA-seq) data were subjected to quality control analyses using FastQC (v0.11.7) (https://www.bioinformatics.babraham.ac.uk/projects/fastqc). Adapter sequence contamination, low quality read regions, and long homopolymeric stretches were removed using TrimGalore (https://github.com/FelixKrueger/TrimGalore). Processed RNA-seq reads (21–70 million reads per sample, average 41 million reads per sample) (Additional file 3) were aligned to the hg38 build (GRCh38.primary_assembly) of the human reference genome using Hisat2 (v2.2.0) (Kim et al. 2019). MultiQC (Ewels et al. 2016) was used for quality control analysis on the resulting bam files by comparing all metrics obtained from Samtools (v1.10) (Danecek et al. 2021), Qualimap (García-Alcalde et al. 2012) and FastQC. A schematic illustration of the analysis steps is provided in Additional file 4.

#### RNA-Seq and differential gene- and transcriptome-expression analysis

We conducted two separate alignments, utilizing indexes provided by the Hisat2 package for both gene and transcript level analysis. For gene expression analysis, read counts for all genes were obtained using FeaturesCounts (Liao et al. 2014). For the differential expression analysis, read counts generated with FeaturesCounts were compared between groups using DESeq2 (Love et al. 2014), within R (v. 4.3.2). Genes with a ≥ 0.5 log2 Fold Change (FC) cutoff were considered differentially expressed. For the transcript-level expression analysis, StringTie (Pertea et al. 2015) was utilized for the assembly and estimation of transcripts abundance. Finally, Ballgown (Frazee et al. 2015) was used for the differential expression analysis of the transcripts, within R (v. 4.3.2). Transcripts with a log_2_ Fold Change (log_2_ FC) ≥ 0.5 were considered as differentially expressed.

#### RNA editing analysis

Raw RNA-seq data were processed following an in-house developed pipeline based on the SPRINT toolkit (Zhang et al. 2017). All potential RNA editing sites were identified using the “sprint main” option within SPRINT, with default parameters. A-to-G and T-to-C mismatches were considered as potential ADAR-mediated RNA editing sites, while C-to-T and G-to-A mismatches were considered as potential Apolipoprotein B mRNA editing enzyme catalytic polypeptide (APOBEC)-mediated RNA editing sites. We applied several quality control analyses, including the assessment of the whole RNA:DNA Difference (RDD) spectrum representation, the estimation of editing event enrichment in *Alu* and other repetitive regions, and the overlap of identified RNA editing sites with RNA editing events deposited in REDIportal v2, which is considered the most annotated and comprehensive RNA Editing database (Karagianni et al. 2023). Custom scripts were used to annotate whether RNA editing sites are located within or near a homopolymer sequence, and the number of times the 100 bp flanking region of the RNA editing site maps to the reference genome with more than 90% sequence similarity as reported by BLAT (Kent 2002). The Open-CRAVAT toolkit (Pagel et al. 2020) was utilized to obtain additional information about the sites where editing events were identified. More specifically, we obtained information from ClinPred (v1.0.0) (Alirezaie et al. 2018), Clinvar (v2024.02.01) (Landrum et al. 2018), Human Phenotype Ontology (v2.1.0) (Köhler et al. 2021), the database for Single Nucleotide Polymorphisms (dbSNP, v154.0.2) (Sherry et al. 2001), the Encyclopedia of DNA elements, transcription factor binding site (ENCODE TFBS, v1.0.2) (The ENCODE Project Consortium 2012), ENSEMBL Regulatory Build (v1.0.3) (Zerbino et al. 2015), the genome Aggregation Database (gnomAD3, v1.1.0) (Karczewski et al. 2020), the microRNA database (miRbase, v1.0.1) (Kozomara and Griffiths-Jones 2014), non-coding RNA (ncRNA, v2022.12.12) (Bao et al. 2015), Pseudogene (v45.0.0) (Harrow et al. 2012) and Repeat Sequences (v2020.10.16) (Haeussler et al. 2019) annotations.

Strict filtering criteria were applied to differentiate between true RNA editing events and false positive results. Samples with low percentage of uniquely mapped reads and high percentage of duplicated reads were excluded from the analysis. Only sites with a minimum base coverage of 10, minimum editing frequency of 10% and minimum quality per base of 25 were considered adequate for our analysis. To reduce false positives, sites located in pseudogenes or sites that were assigned a dbSNP identification (ID) and were not reported in the REDIPortal database were excluded. Sites not in *Alu* regions, located in simple repeat or low complexity regions, were also excluded. RNA editing analysis quality control metrics for the tested samples, confirming high confidence identification of RNA editing events and the validity of the utilized pipeline are presented in Additional file 4.

To determine global RNA editing profiles (editing frequency distribution, actual number of editing events per phenotype group, genomic distribution of RNA editing events in control and ALS-Ox samples) we considered RNA editing events occurring in at least 50% of the samples in each phenotype group. Wilcoxon’s signed-rank test was used to compare the distribution of global RNA editing events levels (% mean editing frequency per editing site) in the control and ALS-Ox groups. Statistical significance was considered for p-value < 0.05. A github repository containing the in-house scripts used is available upon request (https://github.com/Dafoulab/ALS_RNA_Editing_Project).

#### Gene expression RNA editing correlations

For further inspection and visualization of gene expression or RNA editing alterations in selected targets, normalized expression counts or mean editing frequencies at differentially edited (DEdit) sites per sample were plotted and tested (unpaired two-tailed t-test or Mann–Whitney test when deviations from normal distribution were determined following Kolmogorov Smirnov analysis) using the GraphPad Prism software v9.0.0. Statistical significance was considered for p-values < 0.05.

To determine whether ADAR expression levels correlate with global RNA editing levels, Pearson correlation analysis was performed using the GraphPad Prism software v9.0.0, considering normalized gene counts for ADAR1, ADAR2 or ADAR3 expression for each sample and corresponding overall mean editing frequency. Similarly, to determine whether RNA editing alterations correlate with gene expression in selected targets, Pearson correlation analysis was performed considering log_2_ FC of gene expression (DESeq2 analysis) and log_2_ FC of DEdit sites (FC: MeanDEdit frequency ALS-Ox/MeanDEdit frequency Control). To assess the contribution of each DEdit site on gene expression regulation, similar analyses were performed considering editing frequencies at individual sites. Pearson correlation values (r) > 0.5 with p < 0.05 were considered significant.

#### Functional annotation and pathway analysis

To identify molecular processes affected by RNA editing alterations, we focused on DEdit transcripts. The Database for Annotation, Visualization and Integrated Discovery (DAVID, https://david.ncifcrf.gov/summary.jsp, accessed on 11 March 2024) (Huang et al. 2008; Sherman et al. 2022), was used for functional annotation (UP_KW_CELLULAR_COMPONENT) and pathway analysis (Kyoto Encyclopedia of Genes and Genomes-KEGG_PATHWAY) using default settings. Acquired results were plotted according to − log10(p-value) using the GraphPad Prism software v9.0.0.

#### Human autopsy material

The use of human autopsy material in this study has been approved by The Research Ethics and Deontology Committee of the Aristotle University of Thessaloniki (Prot. No. 196519/2021, and Prot. No.: 212186/2021-07/09/2021-for modification by addition of the Edinburgh Brain and Tissue Bank to the biological material provider). Human tissue was provided by the University of Edinburgh Brain and Tissue Bank following pertinent Material Transfer Agreement.

Frontal cortex tissue (Broadmann Area 9) from sporadic ALS and age-matched control donors (n = 3 samples/group) were analyzed. Table 2 provides sample information.

**Table 2:** Human tissue sample information

MRC data base number (BBN)	Sample ID used in this study	Brain region	Condition	Gender	Age (years)
SD021/21	ALS-1	Frontal Cortex (BA9)	ALS	Female	61
SD010/21	ALS-2	Frontal Cortex (BA9)	ALS	Male	65
SD019/17	ALS-3	Frontal Cortex (BA9)	ALS	Male	64
SD030/18	Cntr-1	Frontal Cortex (BA9)	Control (Non-Dementia)	Male	63
SD046/17	Cntr-2	Frontal Cortex (BA9)	Control (Non-Dementia)	Female	65
SD039/14	Cntr-3	Frontal Cortex (BA9)	Control (Non-Dementia)	Male	63

#### RNA editing validation

##### gDNA, RNA extraction and cDNA synthesis

Human tissue samples were processed with the Purelink genomic DNA extraction kit (Invitrogen, Cat No K182001) for genomic DNA (gDNA) extraction and with the RNAsey Lipid Tissue Mini Kit (Qiagen, Cat No 74804) with additional DNAse treatment (Qiagen, RNAse_Free DNAse set, Cat No 79254) for RNA extraction, according to the manufacturers’ instructions. For genomic DNA isolation, 20–25 mg tissue were processed, and DNA was eluted in 100 μl elution buffer in a two-step elution process (100 μl each elution). For RNA extraction, up to 30 mg tissue were processed and RNA was eluted in 30 μl, following a two-step elution process, entailing the reloading of the first eluate on the column. The quantity of extracted nucleic acids (gDNA, RNA) was determined spectrophotometrically (Thermo Scientific, Nanodrop 2000). The quality of RNA was determined by capillary electrophoresis (Agilent, 5300 Fragment Analyzer). The RNA Integrity Number (RIN) values determined were 7.5 ± 0.6 (range 6.8–7.9) for ALS samples and 7.2 ± 1.2 (range 5.9–7.9) for Control samples. Reverse transcription was performed in a final volume of 20 μl, using 1 μg RNA, and both the Random 6mers and oligodT primers included in the PrimeScript™ RT reagent Kit (TAKARA, Cat No RR037A), following manufacturers’ instructions.

##### CACNA1C gDNA and cDNA amplification, PCR purification and sequencing

The intronic region of CACNA1C harboring the differentially edited sites highlighted by our bioinformatics analysis was amplified using the high Fidelity Q5 DNA polymerase (NEB, Cat No M0491L). PCR reactions were performed in a final volume of 50 μl, according to the manufacturer instructions. The following primer set: huCACNA1C_intron3_F: 5′ TCGTCGGCAGCGTCAGATGTGTATAAGAGACAGCCTTTCACAGAGGCAGTTCC 3′ and huCACNA1C_intron3_R: 5′ GTCTCGTGGGCTCGGAGATGTGTATAAGAGACAGGGTCCCCTGGCCTACAATAA 3′, was used to amplify a 427 bp region. Reactions were performed at an annealing temperature of 67 °C. As template, 10 ng cDNA and 50 ng gDNA were used in corresponding amplifications. PCR products were analysed on 2% agarose gels and purified using the PCR clean-up and gel extraction kit (Macherey–Nagel, Cat No 740609.250). Targeted re-sequencing was performed on an Illumina MiSeq instrument, at the Institute of Applied Biosciences at the Centre for Research and Technology Hellas (INAB, CERTH).

Sequencing reads were quality checked using FastQC (v0.11.7) (https://www.bioinformatics.babraham.ac.uk/projects/fastqc) and trimmed at 3′ end to remove adaptor sequence contamination with TrimGalore (https://github.com/FelixKrueger/TrimGalore). The cDNA and gDNA cleaned reads were then independently aligned to the GRCh38 human reference genome using the BWA alignment tool (Li and Durbin 2009) with default parameters. The resulting SAM files were converted to BAM files using the Samtools suite (Danecek et al. 2021) and basic statistics were calculated using Picard tools (“Picard Toolkit.” 2019. Broad Institute, GitHub Repository. https://broadinstitute.github.io/picard/; Broad Institute). Only uniquely mapped reads above mapping quality 20 were utilized for further analysis (MAPQ > 20). All potential RNA Editing Sites (RES) were calculated using JACUSA2 (Piechotta et al. 2022) and additionally checked for artefacts using Integrative Genome Viewer (IGV) (Robinson et al. 2023). Editing frequency cutoff was set to 0.1 (10% editing frequency and above). Duplicate reads as well as vendor failed reads were excluded from the analysis.

##### CACNA1C expression analysis

CACNA1C expression in the tested human autopsy samples was determined through quantitative real-time PCR (qRT-PCR), using the SYBR Fast Universal 2X qPCR Master Mix Kit (Kapa Biosystems, Cat No KK4601) and the 2-ΔΔCt method for relative quantification. GAPDH expression was used for normalization. Reactions were performed under default settings, in an ABI 7500Fast thermal cycler, in a final volume of 20 μl, using 20 ng cDNA and 0.1 μΜ of each primer. The following primer sets were used: hu-CANA1C-qRT-F: 5′ TGATTCCAACGCCACCAATTC 3′, hu-CANA1C-qRT-R: 5′ GAGGAGTCCATAGGCGATTACT 3′ and GAPDH-F: 5′ CAG CCTCAAGATCATCAGCA 3′, GAPDH-R: 5′ TGTGGTCATGAGTCCTTCCA-3′. Correlation analysis between CACNA1C expression and editing levels was performed using the GraphPad Prism software v9.0.0.

## Results

### Global RNA Editing is reduced in the frontal cortex of the ALS-Ox subtype

We determined RNA editing profiles in the frontal cortex of ALS-Ox cases compared to controls. We focused on the frontal cortex because it represents a region involved in the disease that has not been systematically studied in terms of RNA editing. Moreover, considering that the frontal cortex is affected at a later stage during disease progression (Li et al. 2021; Pandya et al. 2022), we reasoned that it would allow the identification of disease-related changes within a more intact tissue background.

**Fig. 1:**
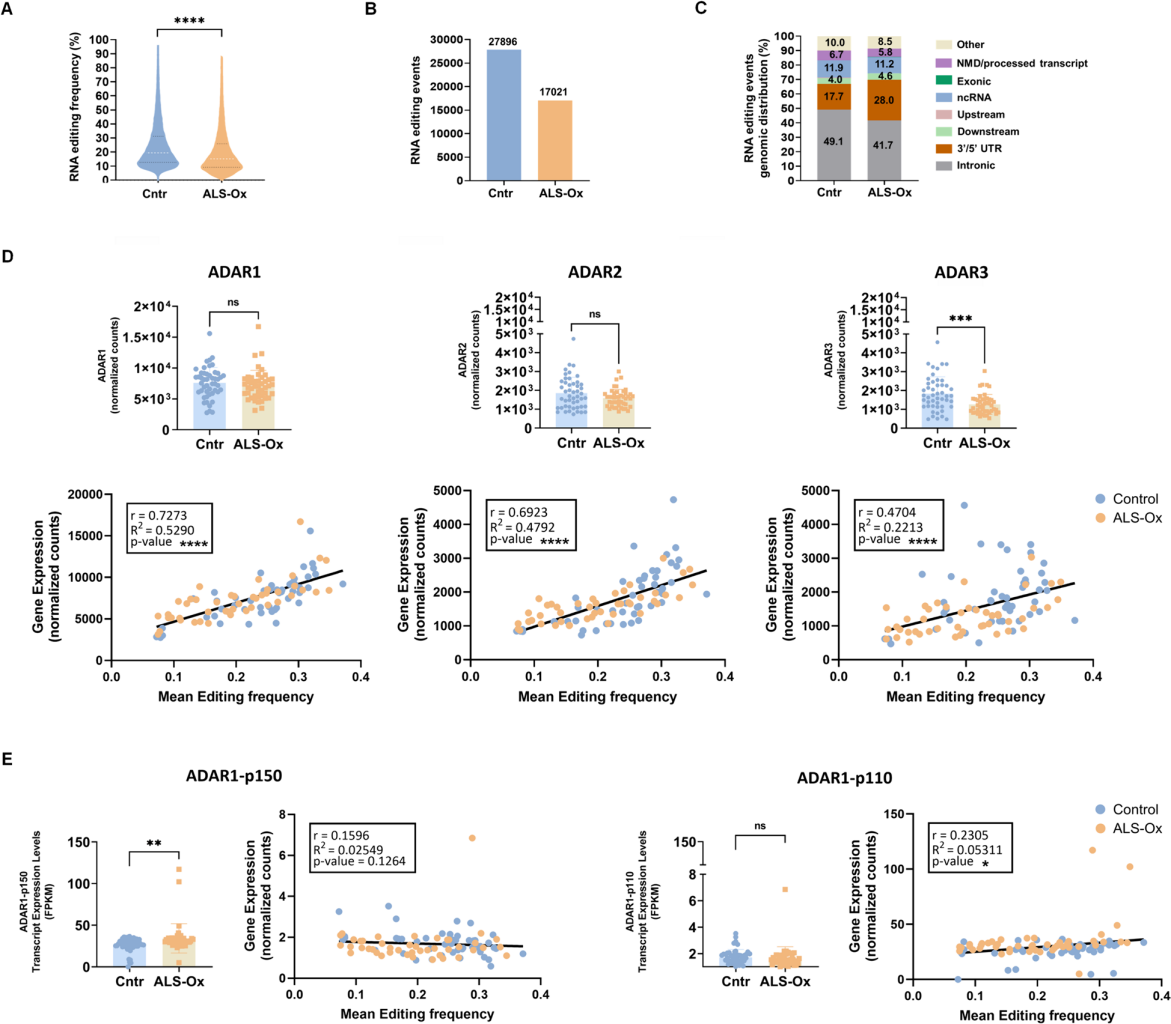
RNA editomes in the frontal cortex of control and ALS-Ox cases. **A** Reduced global RNA editing frequency in ALS-Ox cases compared to controls. Violin plots present the distribution of global RNA editing events levels (% mean editing frequency per editing site) in the control and ALS-Ox groups. The white dotted line indicates the median. Statistical significance was determined by Wilcoxon’s signed-rank test. ****p < 0.001. **B**, **C** Reduced number of RNA editing events (**B**) and altered genomic distribution of RNA editing (**C**) in ALS-Ox cases compared to controls. The bar graph depicts the number of RNA editing events detected per phenotype group. The graphs in **C** present the percent (%) distribution of RNA editing events per genomic region (intronic, 3′/5′ UTR, downstream, upstream, ncRNA, exonic, NMD/processed transcript, other) following the colour code legend on the right. Reduced representation of editing in intronic regions and increased representation in 3’UTRs is observed in ALS-Ox compared to control cases. **D**, **E** ADAR1 and ADAR2 expression levels positively correlate with overall editing frequency. The bar graphs show ADAR1, ADAR2, ADAR3 (**D**), ADAR1-p110 (transcripts ENST00000368471.8; ENST00000649022.2; ENST00000681683.1), ADAR1-p150 (transcript ENST00000529168.2) (**E**) levels in the control and ALS-Ox groups. Statistical significance (unpaired, two-tailed t-test) is denoted by star symbols (ns: non-significant, ***p < 0.001). Below the gene expression graphs we show the corresponding correlation curves between RNA editing levels and each ADAR expression. Data referring to ALS-Ox and Control groups are shown in orange and blue colour respectively. The Pearson correlation (r) value between the corresponding ADAR expression and overall editing levels, the R^2^ value and the corresponding p-values are shown in each case. Statistical significance is denoted by star symbols (****p < 0.0001, *p < 0.05)

We identified high confidence RNA editing events, verified by subsequent quality control analysis (Additional file 4), and established editomes in the frontal cortex of control and ALS-Ox cases (Fig. 1, Additional file 5). We detected a significant reduction of global RNA editing frequency (Fig. 1A) and an overall reduction in the absolute number of RNA editing events (Fig. 1B) in the disease condition. In addition, we observed altered genomic distribution of RNA editing events, characterized by underrepresentation in intronic regions and overrepresentation in 3′ untranslated regions (3′UTRs) in the ALS-Ox cases (Fig. 1C). These findings were replicated following analysis of additional ALS-Ox (n = 17) and control (n = 6) cases frontal cortex RNA-seq data, retrieved from the GSE124439 dataset (Additional file 2A–C). This sample cohort was utilized as a verification cohort in our study (Additional file 2).

To determine whether the observed RNA editing reduction is associated with ADAR1 and/or ADAR2 expression, we assessed ADAR1 and ADAR2 expression levels (Additional file 6). For ADAR1 we additionally determined the expression of its two main isoforms (Additional file 7); the constitutively expressed ADAR1-p110, which is preferentially located in the nucleus, and the interferon-induced ADAR1-p150, which is mainly located in the cytoplasm and exerts immunomodulatory effects, acting as a negative regulator of the melanoma differentiation-associated protein 5-Mitochondrial antiviral-signaling protein (MDA5-MAVS) pathway (Pestal et al. 2015; Li et al 2022). In addition, we assessed the expression of the catalytically inactive ADAR3, known to act as a negative regulator of RNA editing through competitive binding on ADAR1/ADAR2 RNA substrates (Chen et al. 2000; Oakes et al. 2017; Tan et al 2017).

Our gene expression analysis (Additional file 6) did not reveal a statistically significant difference in ADAR1 or ADAR2 mRNA levels between control and ALS-Ox cases (Unpaired, two-tailed t-test p values 0.4548 and 0.0624 respectively), even though we observed a trend towards downregulation in the case of ADAR2. On the other hand, we detected significantly reduced expression of ADAR3 (Mann Whitney test p value 0.0008) (Fig. 1D). Regarding ADAR1 isoforms (Additional file 7), ADAR1-p150 showed increased levels in the disease condition (Mann Whitney test p value 0.0026), suggestive of immune related responses triggering its expression, while no significant changes in ADAR1-p110 levels were observed (Fig. 1E).

Pearson correlation analysis verified positive correlations between overall editing levels per sample and ADAR1 or ADAR2 expression (Pearson correlation r = 0.73, p = 1.5 × 10^–16^ and r = 0.69, p = 1.5 × 10^–14^ respectively), which was stronger for ADAR1. For ADAR3, the correlation value was below 0.5 (r = 0.47, p = 0.000002) (Fig. 1D). Similarly, ADAR1-p110 and ADAR-p150 correlation values were below 0.5 (r = 0.2305 for ADAR1-p110 and r = 0.1596 for ADAR1-p150).

### Differentially edited transcripts are enriched in synapses

**Fig. 2:**
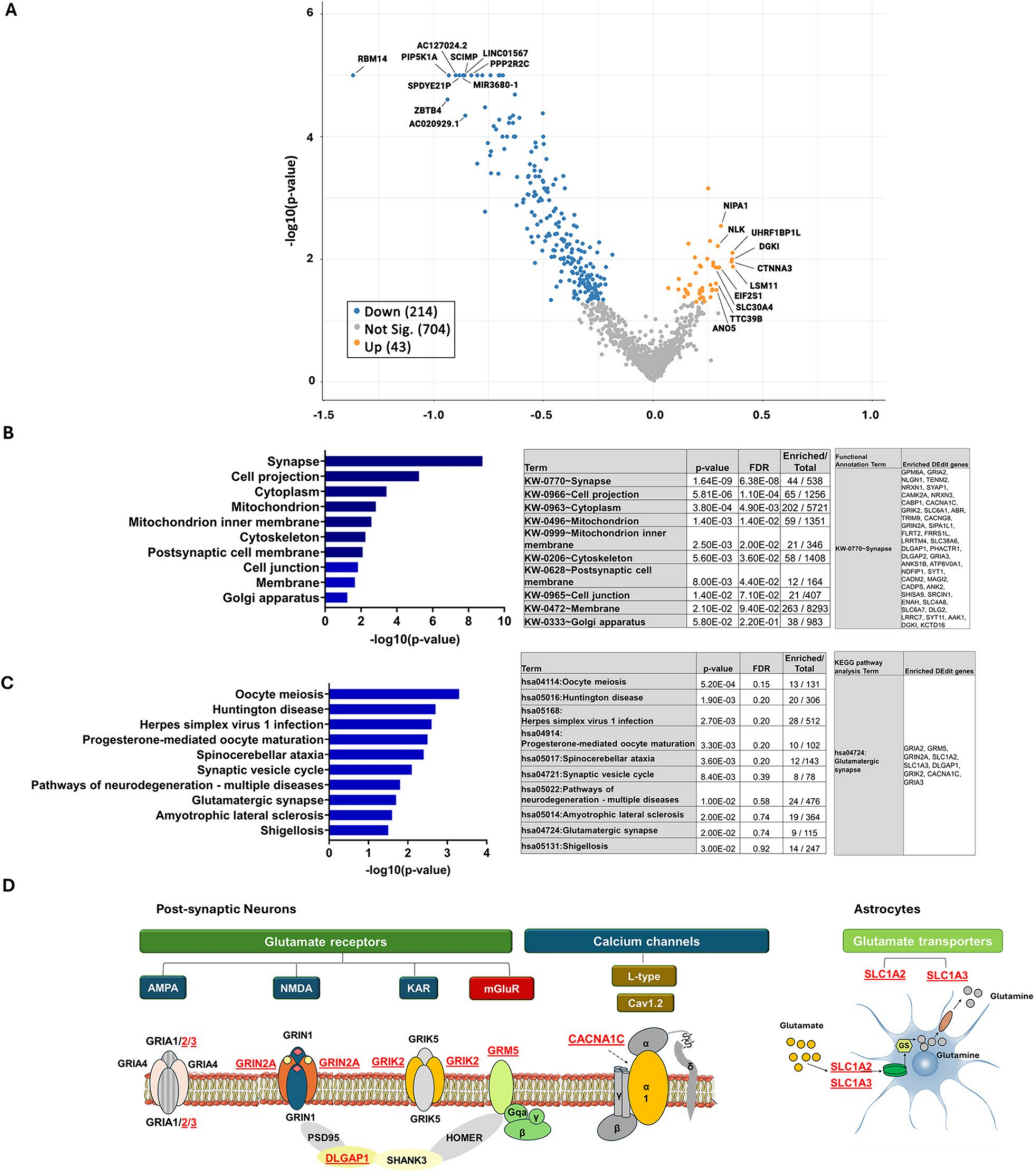
Overview of RNA editing alterations in ALS-Ox samples. **A** Genes presenting RNA editing alterations in ALS-Ox cases relative to controls. The volcano plot depicts the log_2_ FC of RNA editing levels per gene against − log10(p-value) of the corresponding comparison (ALS-Ox versus control samples). Marked are the top 10 genes presenting the highest decrease and top 10 genes presenting the highest increase in editing levels (p-value < 0.05). The total number of genes displaying reduced (214), increased (43) or non-significant editing changes (704) in ALS-Ox cases compared to controls are shown within parentheses in the legend on the left. The majority of genes show reduced editing in the disease condition. **B**, **C** Functional annotation (**B**) and pathway analysis (**C**) on differentially edited transcripts highlights enrichment in the synapse compartment and in pathways associated with synaptic function. The graphs show enrichment, as determined by − log10(p-value), for functional annotation terms (**B**) and KEGG pathways (**C**). The tables list enriched terms along with corresponding p-values and FDR values, and the number of enriched genes (Enriched) against the total number of genes per term (Total). For the terms of interest (synapse in **B**, glutamatergic synapse in **C**) the enriched gene names are also listed. **D** Schematic illustration of DEdit targets enriched in the glutamatergic synapse pathway. DEdit targets are distributed in post-synaptic neurons (GRIA2, GRIA3, GRIN2A, GRIK2, GRM5, CACNA1C, DLGAP1) and astrocytes (SLC1A2, SLC1A3)

We then investigated transcripts presenting editing alterations between control and ALS-Ox cases. Consistent with the overall editing reduction (Fig. 1A), most transcripts displayed significantly reduced mean editing in the disease condition, while for a smaller number of transcripts (~ 16%) increased editing was detected (Fig. 2A). We focused on DEdit transcripts, i.e. transcripts presenting statistically significant difference in terms of RNA editing frequency (Unpaired, two-tailed t-test p < 0.05) in at least one RNA editing site between controls and ALS-Ox samples. We identified 12,395 DEdit sites within 695 transcripts.

To gain insight into the molecular processes affected by the observed RNA editing alterations, we performed functional annotation and pathway analyses using the DEdit transcripts as input. Functional annotation (cellular compartment) using the DAVID software (https://david.ncifcrf.gov/summary.jsp) highlighted synapse as the top hit (p = 1.64E−09, False Discovery Rate (FDR):6.4E−08). In addition, pathways associated with synaptic functions (synaptic vesicle cycle p = 8.4E−03, FDR = 0.39, glutamatergic synapse p = 2.0E−02, FDR = 0.74) were among the ten most enriched terms identified following KEGG pathway analysis (Fig. 2B,C. Additional File 8).

These data indicate functional effects of RNA editing alterations on synaptic (dys)function, suggesting that further study of RNA editing in selected targets may provide a better understanding of synaptic changes in ALS-Ox cases. Considering that the glutamatergic synapse represents the most abundant excitatory synaptic type and that the ‘glutamate hypothesis’, referring to glutamatergic transmission disturbances, has been long proposed as a major pathophysiological mechanism in ALS (Shaw and Ince 1997; Blasco et al. 2014), we focused on the glutamatergic synapse pathway. The glutamatergic synapse pathway associated DEdit transcripts highlighted by our pathway analysis (Fig. 2D), encode for: (a) metabotropic glutamate receptors (GRM5), (b) subunits of α-amino-3-hydroxy-5-methyl-4-isoxazolepropionicacid-AMPA (GRIA2, GRIA3), *N*-methyl-d-aspartate -NMDA (GRIN2A) and Kainate (GRIK2) ionotropic glutamate receptors, (c) calcium channel subunits (CACNA1C) and (d) scaffolding proteins (DLGAP1). In addition, astrocytic enriched/specific transcripts, encoding for glutamate transporters with known effects on synaptic modulation (SLC1A2, SLC1A3, (Rothstein et al. 1995), were also differentially edited.

Similar pathway analyses performed on the verification cohort (GSE124439) DEdit targets, replicated initial analysis findings (Additional file 2, D, E). Functional annotation (cellular compartment) highlighted synapse as the top hit (p = 1.25E−13, False Discovery Rate (FDR): 4.50E−12). Further, a significant enrichment of DEdit transcripts in the glutamatergic synapse pathway (KEGG pathway analysis, p = 3.64E−07, False Discovery Rate (FDR): 1.05E−04) was identified, followed by a similar enrichment in the dopaminergic synapse pathway (p = 1.15E−05, False Discovery Rate (FDR): 0.0016).

### RNA editing modifies glutamatergic synapse transcripts through recoding and gene expression regulation

**Fig. 3:**
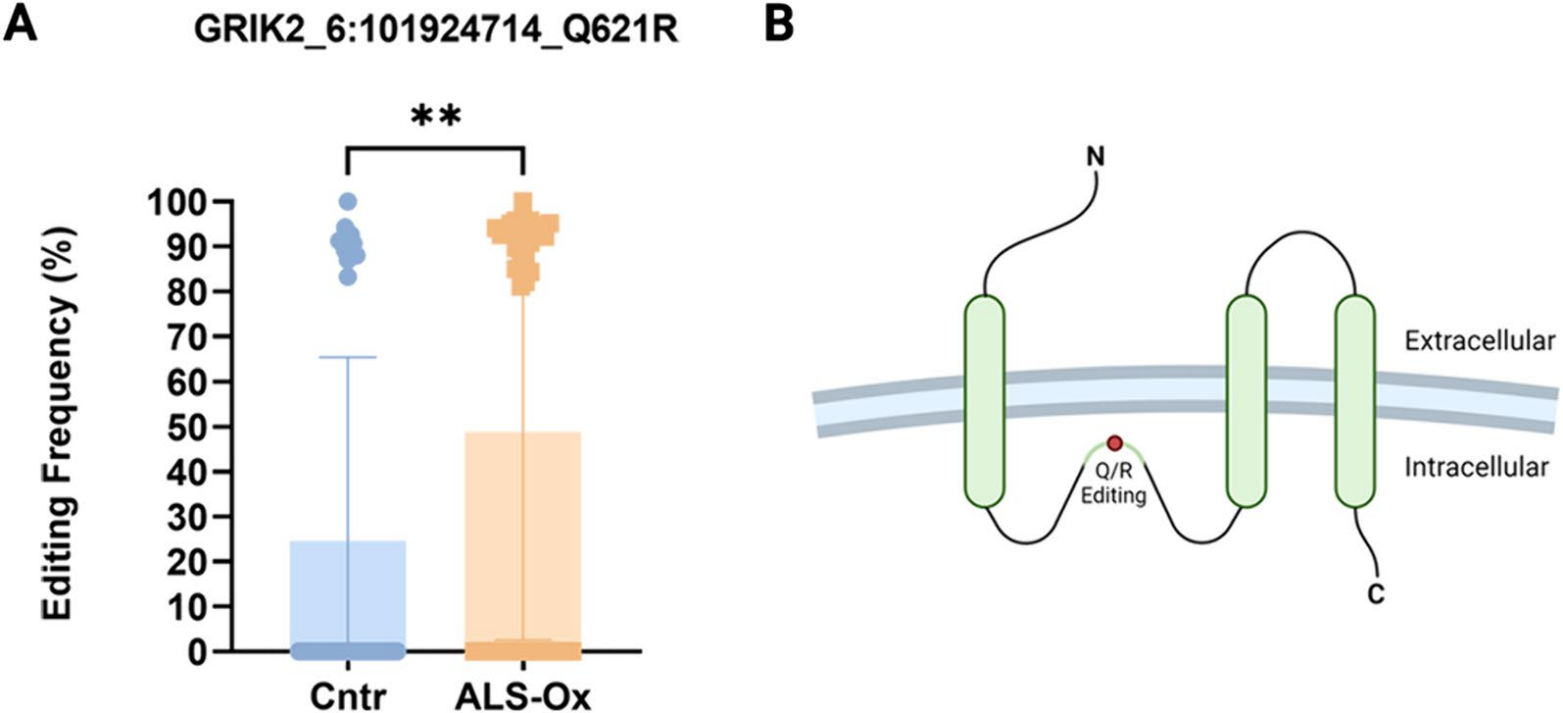
Recoding in the GRIK2 Q621R site in the frontal cortex of ALS-Ox cases. **A** The graph depicts editing frequencies at the Q621R recoding site (6:101924714) in the control and ALS-Ox groups. Statistical significance (unpaired, two-tailed t-test) is indicated (**p < 0.01) **B** Schematic of the recoding site presenting altered editing within the encoded protein. The figure in **B** was created with BioRender.com

To determine functional effects of RNA editing alterations in the glutamatergic synapse enriched DEdit transcripts, we first sought for RNA editing events resulting in amino acid changes (recoding). We detected increased editing at the Q621R site of the GRIK2 (Kainate receptor subunit) transcript (6:101924714, Unpaired, two tailed test, p = 0.0060, Fig. 3), suggesting an impact on the receptor’s function.

We next investigated whether RNA editing changes affect the expression of the targets of interest, by integrating gene expression and RNA editing analyses. Focusing on gene expression first (Additional file 6), we identified a general increase in the expression of glutamate receptor subunits; we also identified transcripts with reduced expression (CACNA1C).

When editing changes were considered, despite the overall editing reduction in the disease, we noticed DEdit transcripts with increased editing and concurrent similar expression changes (GRIA2, GRIA3, GRIK2). GRIA2 and GRIA3 showed modest (log_2_ FC: 0.4–0.5) but significant expression changes (Mann Whitney test p = 0.0032 and 0.0366 respectively) along with significant increase in RNA editing frequency (GRIA2 unpaired t-test p = 0.0171, GRIA3 Mann Whitney test p = 0.0010). GRIK2 also displayed increased editing (Mann Whitney test p = 0.0047), and a trend towards increased expression (log_2_ FC: 0.3, unpaired, two tailed t-test p = 0.38). Among the transcripts displaying reduced editing, a strong downregulation of editing (Mann Whitney test p < 0.0001) and reduced expression (log_2_ FC: − 0.3, Mann Whitney test p < 0.0001) was observed in CACNA1C, while other transcripts showed editing changes that did follow expression.

**Fig. 4:**
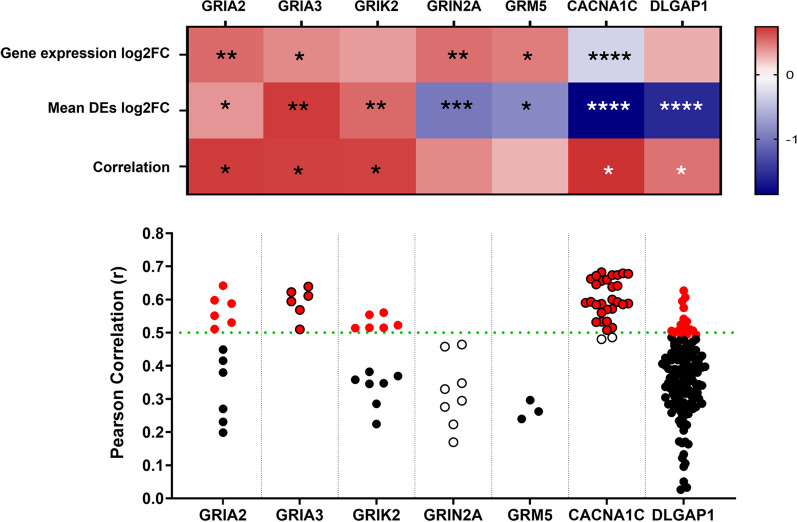
Gene expression, RNA editing patterns and their correlation in glutamatergic synapse enriched DEdit transcripts. The heatmap presents changes in ALS-Ox cases relative to controls referring to gene expression (log_2 _FC expression change) and editing (log_2 _FC mean differential editing changes) following the colour code on the right. Statistical significance (Mann–Whitney or Unpaired two-tailed t-test) is denoted by star symbols (*p < 0.05, **p < 0.01, ***p < 0.001, ****p < 0.0001). The correlation between gene expression and mean DEdit is also shown. The cases for which Pearson correlation values (r) > 0.5 were determined are marked (*). The plot depicts Pearson correlation (r) values determined for each DEdit site in the transcripts of interest. Sites presenting Pearson correlation (r) values > 0.5 are marked in red

Pearson correlation analyses considering mean DEdit frequency and expression, identified transcripts with editing-expression correlations; similar analyses taking into account individual DEdits further assessed independent sites’ contributions (Fig. 4). We highlight positive correlations determined for glutamate receptor (GRIA2, GRIA3) and calcium channel (CACNA1C) subunits, which could represent editing changes driving expression alterations (Fig. 4).

Regarding SLC1A2, SLC1A3, we noticed a strong downregulation of editing in SLC1A2 (Mann Whitney p = 0.0003), but no expression changes (log_2_ FC: 0.15, unpaired, two-tailed t-test, p = 0.53) or adequate correlation between editing and expression (r = 0.402). For SLC1A3, increased or reduced editing in each of the two DEdit sites, resulted in a non-significant overall trend towards reduced editing (Mann Whitney p = 0.35) and a similar trend to reduced expression (log_2_ FC: − 0.12, unpaired, two-tailed t-test, p = 0.05) was observed, with no correlation between editing and expression (r = 0.11).

To further characterize the regions affected by RNA editing alterations, we inspected the distribution of DEdit sites within the transcripts of interest. Intronic editing changes were detected in all cases. Table 3 provides detailed information on the distribution of DEdit sites in the targets of interest and their correlation with gene expression.

**Table 3:** Distribution of DEdit sites within the transcripts of interest and their correlation with expression

Target	Description	Intron	DEdit sites^a^	Region^b^	Editing change^c^	Expression correlation^d^
GRIA2	AMPA receptor subunit	2	3	*Alu*^&^ (*AluSx3*)	↓	No
GRIA2	AMPA receptor subunit	11	3	NR	↑	No
GRIA2	AMPA receptor subunit	12	6	*Alu* (*AluSc/AluJo*)	↑	Yes
GRIA3	AMPA receptor subunit	13	6	NR	↑	Yes
GRIK2	Kainate receptor subunit	4	6	REP^#^ (LTR78B)	↓	No
GRIK2	Kainate receptor subunit	15	6	NR	↑	Yes
GRIN2A	NMDA receptor subunit	2	8	NR/REP (THE1C)	↓	No
GRM5	Group I Metabotropic glutamate receptor	2	1	*Alu* (*AluSq2*)	↓	No
GRM5	Group I Metabotropic glutamate receptor	9	2	*Alu* (*AluSq2*)	↓	No
CACNA1C	L-type voltage-gated calcium channel subunit	3	30	*Alu* (*AluJb*)/ REP (L3)	↓	Yes
DLGAP1	Scaffold protein	7	137	*Alu* (*)	↓	Yes for 8 DEdits (adjacent AluJo/AluJb)
SLC1A2	Glutamate transporter	1	15	*Alu* (**)/ REP (L1PA15)	↓	No
SLC1A3	Glutamate transporter	3	2	*Alu* (*AluSg/AluSq2*)	↑/↓	No

Information on the number and distribution of DEdit sites within the tested targets

NR: non-repetitive region

^a^Number of DEdit sites identified in a specific intron

^b^Characterization of the DEdit site-harbouring region(s) in terms of repetitive elements

^c^Editing change in the disease condition

^d^Yes: denotes DEdit sites correlating with expression (r > 0.5)

^&^*Alu*: *Alu* element. The corresponding *Alu* subfamilies are given within parenthesis

^*^AluSz, AluJr, AluSp, AluSq, AluSq2, AluJb, AluSc, AluJr4, AluSx, AluSx1, AluSc5, AluY, AluSg, AluJo, FLAM-C

^**^AluSx, AluJr, AluSx1, AluJb, AluJo, AluSq2

^#^REP: other repetitive regions. The repetitive element type is given within parenthesis

### Validation of CACNA1C RNA editing alterations

Differential editing within CACNA1C intron 3 and correlation between RNA editing and CACNA1C expression levels, emerged as the most robust association from our bioinformatic analyses on both the initial (Fig. 4) and the verification RNA-seq cohorts (Additional file 2, F). Based on our bioinformatic analyses results, available literature data on CACNA1C function, and previous studies reporting deregulated CACNA1C expression in ALS cases (Aronica et al. 2015; Oliveira et al. 2020), we focused on CACNA1C for RNA editing experimental validation.

**Fig. 5:**
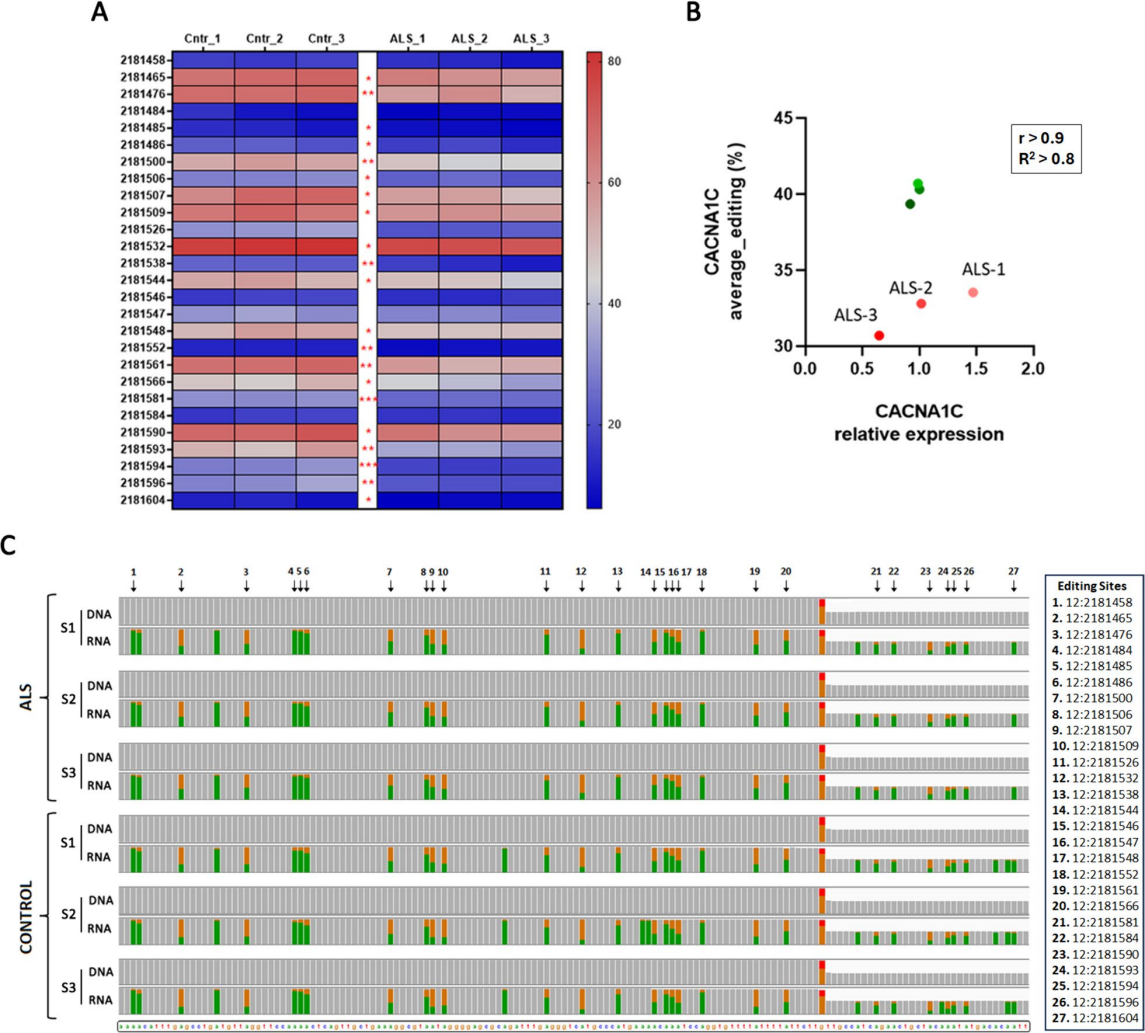
Experimental validation of RNA editing alterations in CACNA1C. **A** The heatmap presents the RNA editing frequency in each editing position, for sites presenting editing frequency above 10% in the tested frontal cortex tissue material from ALS and control cases (n = 3/group). Statistical significance (Mann–Whitney or Unpaired two-tailed t-test) is denoted by star symbols (*p < 0.05, **p < 0.01, ***p < 0.001). **B** Correlation graph between RNA editing and gene expression in the tested ALS and control samples. CACNA1C expression positively correlates with overall editing frequency in ALS and control cases. Data referring to ALS and control groups are shown in red and green colour respectively. The Pearson correlation (r) value between the expression and editing levels in CACNA1C and R^2^ value are shown. **C** Read alignment views of cDNA and corresponding gDNA samples for ALS (ALS-S1, ALS-S2, ALS-S3) and control (CONTROL-S1, CONTROL-S2, CONTROL-S3) cases for the tested region of CACNA1C. Reads are summarized as coverage plots for each sample. Individual base mismatches above 10% are displayed with different colours (green for Adenine-A, orange for Guanine-G, red for Thymine-T). Each arrow represents a validated RNA editing position with altered frequency between ALS and control cases. The actual coordinates of differentially edited sites are shown on the left (Coordinates given relative to the hg38 reference genome). 27 out of 30 differentially edited sites highlighted by our bioinformatics analyses were experimentally validated

Frontal cortex tissue (BA9) from three sporadic ALS and three age- and gender-matched non-dementia control samples, acquired from the Edinburgh Brain and Tissue Bank, were used for validation. Molecular subtype information is not available on the analyzed ALS samples, as the ALS-Ox subtype has been recently described (Tam et al. 2019; Eshima et al. 2023), and no classification of biobank human brain tissue material in relation to this subtype exists, to our knowledge. For RNA editing validation, the CACNA1C region of interest (part of intron 3 harbouring the differentially edited sites) was amplified from cDNA and matching gDNA samples and analysed through deep sequencing on an Illumina MiSeq instrument. This approach enables high read coverage, allowing efficient and sensitive RNA editing identification. Raw sequencing data (14.000–170.000 reads/sample) were aligned against the reference human genome (hg38) for identification and quantification of RNA editing events. Through direct comparison between cDNA and matching gDNA, our experimental data verified RNA editing events in 27 out of the 30 sites highlighted by our bioinformatics analysis. In addition, we identified several differentially edited sites between the tested ALS and control samples, verifying RNA editing alterations in 21 out of the 30 bioinformatically identified positions (Fig. 5A, C).

To further acquire experimental evidence on the effect of RNA editing on CACNA1C expression, we performed correlation analysis between experimentally determined CACNA1C expression and editing levels for the ALS and control groups. Despite the low number of tested samples, we detected high Pearson correlation (r) and R^2^ values, but corresponding p-values did not reach statistical significance (Fig. 5B). The editing-expression correlation was more evident in the ALS group, where CACNA1C expression varied between samples (Fig. 5B).

## Discussion

ALS manifestation is dictated by interconnected molecular processes regulated by complex networks (Mead et al. 2023; Wilson et al. 2023). Analysis and integration of different levels of -omics data, including RNA editing, which represents an epitranscriptomic mechanism of molecular network modulation, is a promising approach for delineation of disease pathogenetic mechanisms.

Existing knowledge on the role of RNA editing in ALS is limited and mainly restricted to the spinal cord, one of the most affected regions in the disease. Previous studies either focused on specific targets (Kawahara et al. 2004; Flomen and Makoff 2011; Yamashita and Kwak 2014) or entailed transcriptome-wide analyses utilizing a relatively low number of samples (n = 6–7 per group, D’Erchia et al. 2017; Moore et al. 2019). In this study, we aimed to extend previous knowledge focusing on the frontal cortex, which is affected at a later disease stage (Pandya et al. 2022) and presents neuronal circuit and synaptic alterations (Henstridge et al. 2018; Brunet et al. 2020; Li et al. 2021). Considering the high heterogenicity of the disease, which may hamper the identification of subtype-specific pathogenetic mechanisms, only samples presenting similar transcriptomic profiles were included in our analysis. We focused on the most prevalent molecular subtype (ALS-Ox), as determined by a previous study (Eshima et al. 2023). Our analysis entailed higher number of samples compared to previous studies, increasing the statistical power of the analysis and strengthening the validity of the results.

We report RNA editing alterations in the frontal cortex of ALS-Ox cases, and enrichment of differentially edited transcripts in synapses, a finding replicated following similar bioinformatics analyses on additional ALS-Ox and control samples (GSE124439 dataset, Tam et al. 2019). We focused our analysis on the glutamatergic synapse pathway, considering that alterations in the glutamatergic system have been long proposed to be involved in the disease (Blasco et al. 2014). Our data highlight modulatory effects of RNA editing alterations on glutamate receptor subunits, mediated through recoding and gene expression regulation. We detected increased recoding at the Q621R site in the GRIK2 transcript (6:101924714), encoding for a subunit of kainate receptors (KARs). RNA editing at this site, mediated by ADAR2, occurs within the pore-lining region of the subunit and renders it Ca^++^ impermeable. In addition, the edited variant displays reduced ability to assemble with other subunits and a higher tendency to accumulate in the endoplasmic reticulum (ER) (Evans et al. 2019), suggesting that increased editing at this site may contribute to reduced neuronal activation. Similar to the interpretation of a previous study in epilepsy (Kortenbruck et al. 2001), a compensatory mechanism against neuronal hyper-excitability in the frontal cortex of ALS-Ox cases could be suggested for the increased Q621R GRIK2 recoding.

We also detected regulatory effects of RNA editing on the expression of the AMPA receptor subunits GRIA2 and GRIA3. We found increased expression of these transcripts, in line with a previous study on sALS frontal cortex (Andrés-Benito et al. 2017), and identified positive correlations between editing and expression changes. Altered editing in GRIA2 and GRIA3 has also been reported in the frontal cortex of C9orf72 ALS/FTD cases (Moore et al. 2019), however an opposite editing change and no expression alterations were identified. This difference may be associated with the genetic background of the samples tested between the two studies, as Moore and colleagues studied C9orf72 positive cases, whereas our analysis focused on non-C9orf72 cases (C9orf72 negative: 39, unknown:6). Considering that neuronal excitability is determined by the cooperative action of different receptor types and their subunits (Di Maio 2021), the precise functional effects of RNA editing and subsequent expression alterations on GRIA2 and GRIA3 reported here, can only be determined through functional analyses in appropriate disease systems.

We found a robust positive correlation between reduced editing and expression of CACNA1C, also replicated in the verification dataset, and we experimentally validated RNA editing alterations utilizing frontal cortex human autopsy material from ALS and control cases. Our experimental data validate editing in several positions within CACNA1C and verify differential editing in 27 out of the 30 positions highlighted by bioinformatics analysis. Moreover, our data provides evidence further supporting the editing-expression correlation highlighted by our bioinformatics analysis.

CACNA1C encodes the pore-forming subunit of the L-type (long lasting) voltage-gated calcium channel (VGCC) Cav1.2. Cav1.2 channels are predominantly located in post-synaptic neurons, and in addition to their role on neuronal excitability, they modulate gene expression (Catterall 2011; Simms and Zamponi 2014), mitochondrial function (Hotka et al. 2020) and local translation (Kabir et al. 2017). Altered CACNA1C expression has been reported in the motor cortex of a sALS subtype (Aronica et al. 2015) and in fALS models (Chang and Martin 2016; Oliveira et al. 2020) presenting opposite expression patterns. Different outcomes resulted from the use of L-type channel blockers (Tran et al. 2014) or agonists (Armstrong and Drapeau 2013) in distinct disease models, indicating that CACNA1C is involved in the disease in a subtype-specific manner. This data in light of our findings, reported here, suggests that further studies on CACNA1C may contribute to a better understanding of disease pathogenetic mechanisms, enhancing novel intervention approaches.

We identified RNA editing alterations in intronic regions, suggesting a contribution to RNA processing dysfunctions, a well-accepted disease hallmark (Butti and Patten 2019). RNA editing may affect splicing through several mechanisms entailing modification of *cis*-regulatory elements or induction of structural changes that enable of preclude *trans*-acting regulation (Hsiao et al. 2018; Hu et al. 2022), that could result in aberrant splicing such as activation of cryptic exons and/or polyadenylation signals, which in turn may affect expression. In addition, recent studies highlight a role of ADAR1/ADAR2 mediated RNA editing in inhibition of circular RNAs biogenesis because of double-strand RNA disruption due to editing in intronic *Alu* repeats (Wang et al. 2023b; Kokot et al. 2022). Considering the regulatory effects exerted by circular RNAs (D’Anca et al. 2022) and the accumulating evidence on their deregulation in ALS (Aquilina-Reid et al. 2022; Tsitsipatis, et al. 2022), the investigation of RNA editing effects on circular RNA biogenesis/function is anticipated to provide significant insights into disease pathogenesis. Comprehensive studies on the effects of RNA editing events on splicing and circular RNAs require the development of specialized bioinformatics analysis pipelines and represent future aims of our research. Our data also highlighted altered distribution of RNA editing events in 3′UTR regions and differential editing in 3′UTRs was detected in several synapse enriched transcripts. Considering that 3’UTRs are directly involved in transcript levels regulation through *cis*-acting elements that dictate interactions with miRNA and/or RNA Binding Proteins (RBPs), analysis of miRNA and/or RBP binding sites/motifs affected by RNA editing alterations is expected to allow further mechanistic insights. However, according to our view, the study of RNA editing effects on miRNA binding requires a more comprehensive analysis, utilizing specialized bioinformatics analyses pipelines that combine tools for prediction of RNA editing-mediated miRNA binding motif alteration with experimentally validated miRNA–mRNA interactions and miRNA alterations in the disease context. These approaches are expected to provide additional insights into disease pathogenesis and represent one of our future research aims.

Functional analyses in disease models recapitulating characteristics of the ALS-Ox subtype, focusing on the targets highlighted by our study, are required for the determination of the precise role of RNA editing alterations.

## Conclusions

In conclusion, this study provides for the first time, evidence of widespread RNA editing alterations in the frontal cortex of an ALS molecular subtype, highlighting the involvement of RNA editing in the modulation of transcripts involved in the glutamatergic synapse pathway. We report altered recoding and RNA editing-mediated expression changes in disease-related targets, and further experimentally validate RNA editing alterations in CACNA1C, thus providing targets for further functional studies in disease relevant systems, anticipating to enable a better understanding of disease mechanisms and promote more efficient therapeutic approaches.

### Supplementary Information

Additional file 1. RNA-seq post-mortem sample data. Detailed information on the samples corresponding to the RNA-seq data from the GSE153960 dataset analysed in this study.

Additional file 2. Summary of the characteristics and analysis results of the verification cohort used in this study. The Table provides clinical and genetic data information on the ALS-Ox and control samples that were retrieved from the GSE124439 dataset and utilized for the analysis. The Figure summarizes bioinformatic analysis results, which replicate the GSE153960 analysis findings.

Additional file 3. Sequencing data and quality control metrics (GSE153960 analysis). Detailed information on the sequencing data and corresponding quality control metrics.

Additional file 4. Analysis pipeline and RNA editing quality control metrics. Schematic illustration of the analysis pipeline utilized in this study and RNA editing analysis quality control metrics verifying high confidence RNA editing events identification and highlighting the validity of the utilized pipeline.

Additional file 5. RNA editing analysis_SPRINT results (GSE153960 analysis). List of the RNA editing events identified following SPRINT analysis per sample and phenotype group.

Additional file 6. Gene expression analysis (GSE153960 analysis). The file provides gene expression analysis data (differential analysis and normalized counts).

Additional file 7. Transcript expression analysis (GSE153960 analysis). The file provides transcript analysis data (differential analysis and normalized counts).

Additional file 8. Functional annotation and pathway analysis (GSE153960 analysis).

## Data Availability

Data analysed in this study are included in the Gene Expression Omnibus (GEO) repository under the accession GSE153960, https://www.ncbi.nlm.nih.gov/geo/query/acc.cgi?acc=GSE153960 and are included in the following published study: Prudencio et al. (2020). Details on the subset of samples analysed in this study are provided in Additional file 1 of this manuscript. Similarly, data analysed for verification purposes are included in the Gene Expression Omnibus (GEO) repository under the accession GSE124439, https://www.ncbi.nlm.nih.gov/geo/query/acc.cgi?acc=GSE124439 and are included in the following published study: Tam et al. (2019). The code used for the analysis is available upon request (github link: https://github.com/Dafoulab/ALS_RNA_Editing_Project).

## Abbreviations

ALS: Amyotrophic Lateral Sclerosis
ALS-Ox: ALS subtype presenting characteristics of oxidative and proteotoxic stress
fALS: Familial ALS
sALS: Sporadic ALS
A-to-I editing: Adenosine to Inosine RNA editing
ADAR: Adenosine Deaminase Acting on RNA
miRNAs: MicroRNAs
ALS/FTD: Amyotrophic Lateral Sclerosis/Frototemporal Dementia
NYGC: New York Genome Center
MND: Motor Neuron Disease
RNA-seq: RNA sequencing
FC: Fold change
APOBEC: Apolipoprotein B mRNA editing enzyme catalytic polypeptide
RDD: RNA:DNA Difference
dbSNP: Database for Single Nucleotide Polymorphisms
ENCODE TFBS: Encyclopedia of DNA elements transcription factor binding site
gnomAD3: Genome aggregation Database
miRbase: The microRNA database
ncRNA: Non-coding RNA
ID: Identification
DEdit: Differentially edited
DAVID: Database for Annotation, Visualization and Integrated Discovery
FDR: False Discovery Rate
KEGG: Kyoto Encyclopedia of Genes and Genomes
3′UTR: 3′ Untranslated region
MDA5-MAVS pathway: Melanoma differentiation-associated protein 5-Mitochondrial antiviral-signaling protein pathway
AMPA: α-Amino-3-hydroxy-5-methyl-4-isoxazolepropionic acid receptor
NMDA: *N*-Methyl-d-aspartate
KAR: Kainate receptor
ER: Endoplasmic reticulum
VGCCs: Voltage-gated calcium channels
