# Supplementary material for: RNA editing regulates glutamatergic synapses in the frontal cortex of a molecular subtype of Amyotrophic Lateral Sclerosis
Source: Mol Med. 2024 Jul 12;30:101. doi: 10.1186/s10020-024-00863-2 (PMC11241978; doi:10.1186/s10020-024-00863-2)
Supplement: Supplementary file 2 — Additional file 2. Summary of the characteristics and analysis results of the verification cohort used in this study. The Table provides clinical and genetic data information on the ALS-Ox and control samples that were retrieved from the GSE124439 dataset and utilized for the analysis. The Figure summarizes bioinformatic analysis results, which replicate the GSE153960 analysis findings. [file 10020_2024_863_MOESM2_ESM.pdf]

ALS cases Clinical Data (GSE124439)								
**ALS molecular subtype (based on Tam et al 2019)**	**Gender**	**Age at onset (years)**	**Site of Motor Onset**	**Family History of ALS/FTD**	**Age at death (years)**	**Disease Duration (months)**	**Subject Group**	**Cause of Death**
ALS-Ox: n=17	Female: 10<br>Male: 7	Range:<br>55-79<br>Average ± SD:<br>63.9 ± 7.6<br>(NA: n=2)	Limb: n= 10<br>Bulbar: n=4<br>Bulbar and Limb: n=1<br>NA: n= 2	Yes: n=2<br>No: n= 1<br>NA: n=14	Range:<br>53-80<br>Average ± SD:<br>67.9 ± 7.1	Range:<br>12-156<br>Average ± SD:<br>58.5 ± 43.8<br>(NA: n=2)	ALS Spectrum MND*: n= 15<br>ALS Spectrum MND, Other Neurological Disorders**: n= 2	ALS: n= 8<br>Respiratory$^{\$}$: n= 3<br>NA: n= 6
**ALS cases available genetic data**								
**SOD1 mutation**	**C9orf72 expansions**	**ATXN2 expansions**						
Negative: n= 17	Negative: n= 17	Negative:<br>n= 16<br>Intermediate (30-33): n= 1						
**Control data (GSE124439)**								
**Non-neurological**	**Gender**	**Age at death (years)**	**Cause of Death**					
Control: n=6	Female: n= 5 Male: n=1	Range:<br>22 - 90<br>Average ± SD:<br>63 ± 23.8	Cardiovascular: n=1<br>Neoplastic: n=2<br>Respiratory: n=2<br>Gastrointestinal: n=1					

MND: Motor Neuron Disease
NA: not available/unknown
* Classical/Typical ALS
** Classical/Typical ALS, Dementia with Lewy Bodies (DLB) (n=1) & Classical/Typical ALS, Alzheimer's Disease (AD) (n=1)
$^{\$}$ Respiratory Failure (n=2), Pneumonia (n=1)

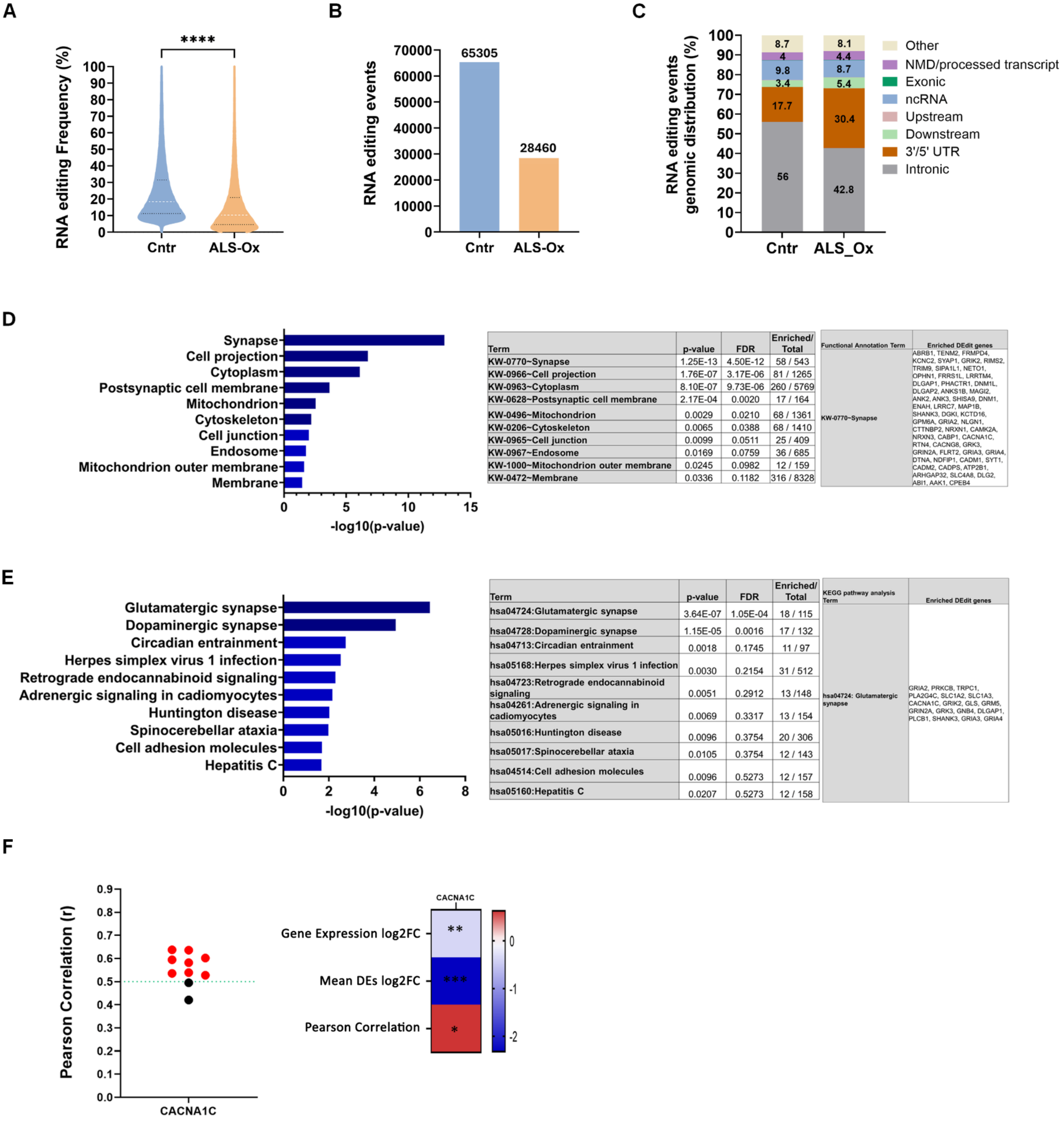

Term	p-value	FDR	Enriched/ Total
KW-0770~Synapse	1.25E-13	4.50E-12	58 / 543
KW-0966~Cell projection	1.76E-07	3.17E-06	81 / 1265
KW-0963~Cytoplasm	8.10E-07	9.73E-06	260 / 5769
KW-0628~Postsynaptic cell membrane	2.17E-04	0.0020	17 / 164
KW-0496~Mitochondrion	0.0029	0.0210	68 / 1361
KW-0206~Cytoskeleton	0.0065	0.0388	68 / 1410
KW-0965~Cell junction	0.0099	0.0511	25 / 409
KW-0967~Endosome	0.0169	0.0759	36 / 685
KW-1000~Mitochondrion outer membrane	0.0245	0.0982	12 / 159
KW-0472~Membrane	0.0336	0.1182	316 / 8328

Functional Annotation Term	Enriched DEdit genes
KW-0770~Synapse	ABRB1, TENM2, FRMPD4, KCNC2, SYAP1, GRIK2, RIMS2, TRIM9, SIPA1L1, NETO1, OPHN1, FRRS1L, LRRTM4, DLGAP1, PHACTR1, DNM1L, DLGAP2, ANKS1B, MAGI2, ANK2, ANK3, SHISA9, DNM1, ENAH, LRRC7, MAP1B, SHANK3, DGKI, KCTD16, GPM6A, GRIA2, NLGN1, CTTNBP2, NRXN1, CAMK2A, NRXN3, CABP1, CACNA1C, RTN4, CACNG8, GRK3, GRIN2A, FLRT2, GRIA3, GRIA4, DTNA, NDFIP1, CADM1, SYT1, CADM2, CADPS, ATP2B1, ARHGAP32, SLC4A8, DLG2, ABI1, AAK1, CPEB4

Term	p-value	FDR	Enriched/ Total
hsa04724:Glutamatergic synapse	3.64E-07	1.05E-04	18 / 115
hsa04728:Dopaminergic synapse	1.15E-05	0.0016	17 / 132
hsa04713:Circadian entrainment	0.0018	0.1745	11 / 97
hsa05168:Herpes simplex virus 1 infection	0.0030	0.2154	31 / 512
hsa04723:Retrograde endocannabinoid signaling	0.0051	0.2912	13 /148
hsa04261:Adrenergic signaling in cadiomyocytes	0.0069	0.3317	13 / 154
hsa05016:Huntington disease	0.0096	0.3754	20 / 306
hsa05017:Spinocerebellar ataxia	0.0105	0.3754	12 / 143
hsa04514:Cell adhesion molecules	0.0096	0.5273	12 / 157
hsa05160:Hepatitis C	0.0207	0.5273	12 / 158

KEGG pathway analysis Term	Enriched DEdit genes
hsa04724: Glutamatergic synapse	GRIA2, PRKCB, TRPC1, PLA2G4C, SLC1A2, SLC1A3, CACNA1C, GRIK2, GLS, GRM5, GRIN2A, GRK3, GNB4, DLGAP1, PLCB1, SHANK3, GRIA3, GRIA4

RNA editomes in the frontal cortex of control and ALS-Ox cases (cohort GSE124439). A. Reduced global RNA editing frequency in ALS-Ox cases compared to controls. Violin plots present the distribution of global RNA editing events levels (% mean editing frequency per editing site) in the control and ALS-Ox groups. The white dotted line indicates the median. Statistical significance was determined by Wilcoxon's signed-rank test. **** p<0.001. **B-C.** Reduced number of RNA editing events (**B**) and altered genomic distribution of RNA editing (**C**) in ALS-Ox cases compared to controls. The bar graph depicts the number of RNA editing events detected per phenotype group. The graphs in **C** present the percent (%) distribution of RNA editing events per genomic region (intronic, 3'/5' UTR, downstream, upstream, ncRNA, exonic, NMD/processed transcript, other) following the colour code legend on the right. Reduced representation of editing in intronic regions and increased representation in 3'UTRs is observed in ALS-Ox compared

to control cases. **D-E**. Functional annotation (**D**) and pathway analysis (**E**) on differentially edited transcripts highlights enrichment in the synapse compartment and in pathways associated with synaptic function. The graphs show enrichment, as determined by -log10(p-value), for functional annotation terms (**D**) and KEGG pathways (**E**). The tables list enriched terms along with corresponding p-values and FDR values, as well as the number of enriched genes (Enriched) against the total number of genes per term (Total). For the terms of interest (synapse in **D**, glutamatergic synapse in **E**) the enriched gene names are also listed. **F.** Correlation between RNA editing in CACNA1C and corresponding expression levels. The plot depicts Pearson correlation (r) values determined for each DEdit site in CACNA1C transcript. Sites presenting Pearson correlation (r) values > 0.5 are marked in red. The heatmap presents changes in ALS-Ox cases relative to controls referring to gene expression (log2FC expression change) and editing (log2FC mean differential editing changes) following the colour code on the right. Statistical significance (Mann-Whitney or Unpaired two-tailed t-test) is denoted by star symbols (*: p <0.05, **: p < 0.01, ***: p< 0.001, ****: p< 0.0001). The Pearson correlation between gene expression and mean DEdit is also shown.
