# Supplementary material for: RNA editing regulates glutamatergic synapses in the frontal cortex of a molecular subtype of Amyotrophic Lateral Sclerosis
Source: Mol Med. 2024 Jul 12;30:101. doi: 10.1186/s10020-024-00863-2 (PMC11241978; doi:10.1186/s10020-024-00863-2)
Supplement: Supplementary file 4 — Additional file 4. Analysis pipeline and RNA editing quality control metrics. Schematic illustration of the analysis pipeline utilized in this study and RNA editing analysis quality control metrics verifying high confidence RNA editing events identification and highlighting the validity of the utilized pipeline. [file 10020_2024_863_MOESM4_ESM.pdf]

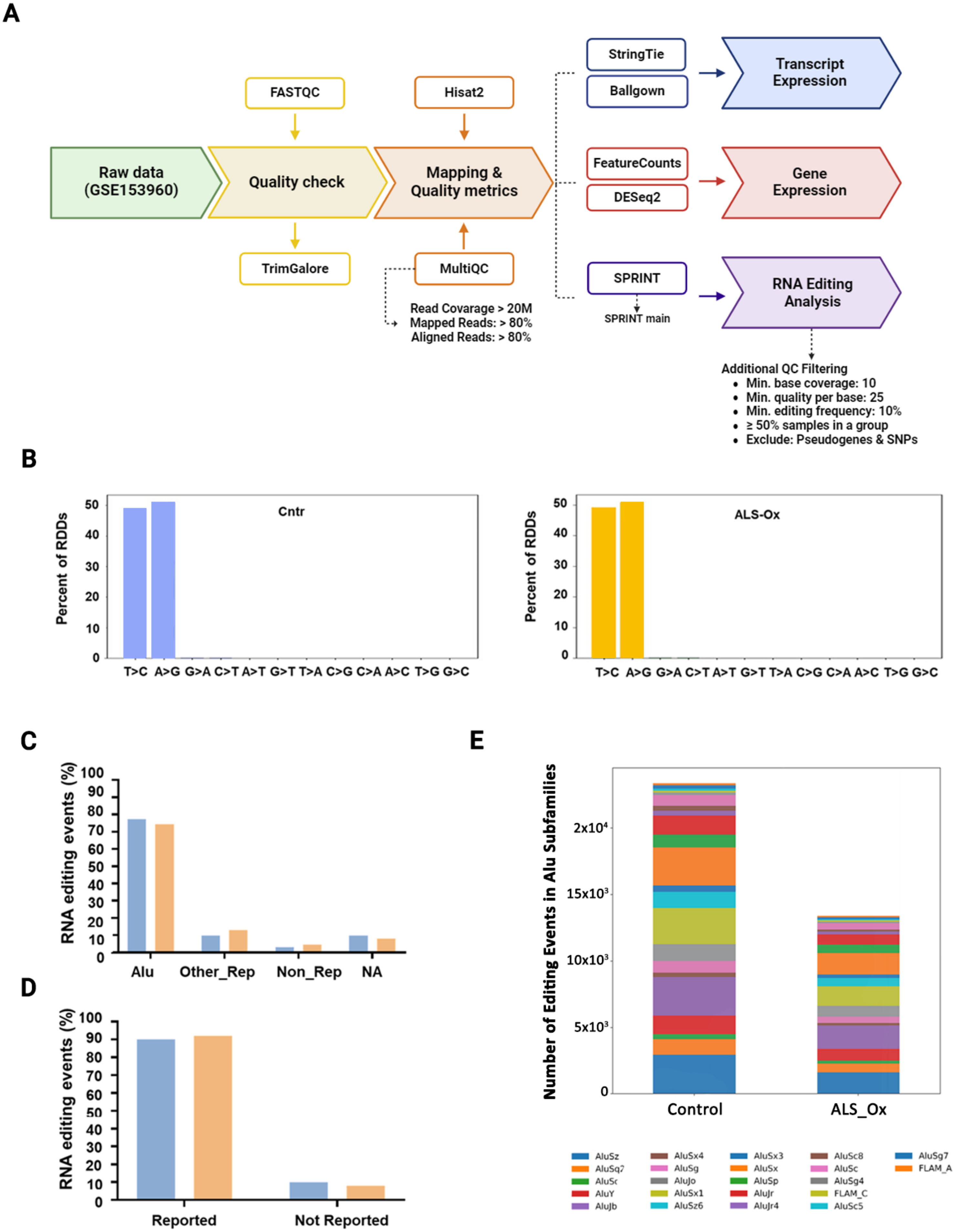

A
FASTQC
Hisat2
StringTie
Ballgown
Transcript Expression
Raw data (GSE153960)
Quality check
Mapping & Quality metrics
FeatureCounts
DESeq2
Gene Expression
TrimGalore
MultiQC
SPRINT
SPRINT main
RNA Editing Analysis
Read Covarage > 20M
Mapped Reads: > 80%
Aligned Reads: > 80%
Additional QC Filtering
Min. base coverage: 10
Min. quality per base: 25
Min. editing frequency: 10%
≥ 50% samples in a group
Exclude: Pseudogenes & SNPs
B
Cntr
ALS-Ox
Percent of RDDs
0
10
20
30
40
50
T>C A>G G>A C>T A>T G>T T>A C>G C>A A>C T>G G>C
C
RNA editing events (%)
0
10
20
30
40
50
60
70
80
90
100
Alu
Other_Rep
Non_Rep
NA
D
RNA editing events (%)
Reported
Not Reported
E
Number of Editing Events in Alu Subfamilies
0
5x10³
10x10³
15x10³
2x10⁴
Control
ALS_Ox
AluSz
AluSq7
AluSc
AluY
AluJb
AluSx4
AluSg
AluJo
AluSx1
AluSz6
AluSx3
AluSx
AluSp
AluJr
AluJr4
AluSc8
AluSc
AluSg4
FLAM_C
AluSc5
AluSg7
FLAM_A

Analysis pipeline and RNA editing quality control metrics. The analysis pipeline utilized in this study **(A)** and RNA editing quality control metrics for the analysed samples **(B-E)**. **A.** Following initial quality control (FASTQC), trimming (TrimGalore) and alignment (HISAT2) to the human reference genome (hg38) was conducted. Samples with >20 million reads and mapping/alignment rates >80% were processed. Feature counts and DESeq2 were utilized for gene expression analysis, while StringTie and Ballgown were used for transcript analysis. SPRINT was used for RNA editing analysis. Events detected in 50% of the samples in the control or in the ALS-Ox group were considered to ensure high confidence events identification. Additional quality control filtering entailed exclusion of pseudogenes and known SNPs. Sites with minimum base coverage 10, minimum quality per base 25 and minimum editing frequency of 10% were considered. **B.** Distribution of all 12 types of potential RNA:DNA differences (RDDs) detected by SPRINT. In line with existing knowledge that ADARs mediate the prevalent RNA editing type resulting in A>G (or T>C reverse strand) RDDs, a high representation of A>G and T>C mismatches was observed. **C.** Distribution of RNA editing events detected by SPRINT in repetitive and non-repetitive regions. High representation in Alu regions was detected, in accordance to existing knowledge. **D.** Distribution of RNA editing events in relevance to events reported in the REDIportal database, corresponding to the most updated and comprehensive database of RNA editing events. Significant overlaps support the validity of the utilized pipeline. **E.** Distribution of RNA editing events in different Alu subfamilies in the control and ALS-Ox cases.
